# Tide as Steering Factor in Structuring Archaeal and Bacterial Ammonia-Oxidizing Communities in Mangrove Forest Soils Dominated by *Avicennia germinans* and *Rhizophora mangle*

**DOI:** 10.1007/s00248-017-1091-y

**Published:** 2017-10-23

**Authors:** Magalí S. Marcos, Anthony D. Barboza, Rosalinde M. Keijzer, Hendrikus J. Laanbroek

**Affiliations:** 1 Department of Microbial Ecology, Netherlands Institute of Ecology (NIOO-KNAW), P.O. Box 50, 6700 AB Wageningen, the Netherlands; 2 Laboratorio de Microbiología y Biotecnología, Instituto Patagónico para el Estudio de los Ecosistemas Continentales (IPEEC, CONICET), Puerto Madryn, Argentina; 3 Centro Interdisciplinar de Pesquisas em Biotecnologia – CIP-Biotec, Universidade Federal do Pampa, Campus São Gabriel, São Gabriel, Brazil; 4 Ecology and Biodiversity Group, Department of Biology, Utrecht University, Utrecht, the Netherlands

**Keywords:** AOA, AOB, Microbial community structure, Mangroves, *Avicennia germinans*, *Rhizophora mangle*

## Abstract

Mangrove species are adapted to grow at specific zones in a tidal gradient. Here we tested the hypothesis that the archaeal and bacterial ammonia-oxidizing microbial communities differ in soils dominated by the mangrove species *Avicennia germinans* and *Rhizophora mangle*. Two of the sampling locations were tidal locations, while the other location was impounded. Differences in the community compositions of ammonia-oxidizing archaea (AOA) and bacteria (AOB) were analyzed by denaturing gradient gel electrophoresis (DGGE) of *amoA* genes and by MiSeq 16S rRNA gene-sequencing. The abundances of AOA and AOB were established by quantitative PCR of *amoA* genes. In addition, we analyzed the total microbial community composition based on 16S rRNA genes and explored the influence of soil physicochemical properties underneath *Avicennia germinans* and *Rhizophora mangle on* microbial communities. AOA were always more abundant than AOB, but the effect of mangrove species on total numbers of ammonia oxidizers was location-specific. The microbial communities including the ammonia oxidizers in soils associated with *A. germinans* and *R. mangle* differed only at the tidal locations. In conclusion, potential site-specific effects of mangrove species on soil microbial communities including those of the AOA and AOB are apparently overruled by the absence or presence of tide.

**Electronic supplementary material:**

The online version of this article (10.1007/s00248-017-1091-y) contains supplementary material, which is available to authorized users.

## Introduction

Mangrove forests are highly valuable ecosystems for the numerous products and fundamental services they provide [1]. These ecosystems are confined to intertidal coastal areas from (sub)tropical regions, and within these areas they often show zonation patterns, in which monospecific bands of trees are formed parallel to the shoreline [2]. In Florida, *Rhizophora mangle* usually occurs lower in the intertidal zone than *Avicennia germinans* that can be found more at in-land sites where tidal inundation is less frequent [3]. Species of the genera *Rhizophora* and *Avicennia*, which are among the most widely distributed mangroves of the world [4], differ in their potential for growth, resource acquisition, stress tolerance, and susceptibility to herbivores [5]. *Avicennia* and *Rhizophora* also differ in their adaptations to live under flooded conditions. One of these adaptations concerns their root systems through which they influence the biogeochemistry of the soil. Distinct differences in soil pH values, redox potentials, sulfide, and organic matter concentrations were reported in soils covered with these mangrove trees [6]. In addition, these species differ in their tissue chemistry: *Rhizophora* spp. have higher contents of total soluble phenolics (including tannins) and higher C:N ratios (i.e., lower nutritive value) than *Avicennia* spp. [5, 7]. These attributes may in turn be associated with the generally slower rates of organic matter decomposition of *Rhizophora* tissues [8].

Ammonia-oxidizing archaea (AOA) of the Thaumarchaeota and ammonia-oxidizing bacteria (AOB) of the β- and γ-Proteobacteria are critical players in the global nitrogen cycle, because they perform the first and often rate-limiting step of nitrification under oxic conditions—i.e., the oxidation of ammonia to nitrite [9, 10]. Since the enzyme ammonia monooxygenase catalyzes this first step in aerobic ammonia oxidation, the *amoA* gene encoding the α subunit of this enzyme has been frequently used as molecular marker in ecological studies of these microorganisms [11]. Culture-independent studies based on *amoA* and on 16S rRNA genes demonstrated a ubiquitous distribution of AOB and AOA in a variety of environments (even extreme habitats) [12], in which their relative abundance and community composition may be determined not only by one factor but by a combination of several environmental characteristics [13]. However, in spite of their widespread distribution, it was only recently that studies focused on AOA and AOB from mangrove ecosystems, and on the environmental factors that determine their abundance and community composition [14–21].

Mangrove trees can have different impacts on soil archaeal and bacterial communities through alteration of the soil microenvironment such as changes in the content of organic matter or other sediment characteristics [22, 23], root exudates [24, 25], and litter inputs [26]. Moreover, recent studies suggested that mangrove plants could be important factors controlling the activity, abundance, and community structure of ammonia oxidizers [14, 15, 18]. Laanbroek et al. observed that three *A. germinans* habitats from Florida that differed in tree height and density of canopy cover also differed in their AOB community composition, suggesting that the environmental factors that control the growth and coverage of *A. germinans* also affect the community composition of AOB [14]. In mangroves from China, the mangrove *Kandelia obovata* promoted higher nitrification rates, and higher abundance of bacterial *amoA* genes and archaeal *amoA* transcripts compared to bare sediments [18]. In this study, numbers of archaeal *amoA* genes and transcripts always exceeded those of their bacterial equivalents. Higher abundances of bacterial than archaeal *amoA* genes and transcripts were also detected in mangrove sediments from Mai Po Nature Reserve (Hong Kong), and in sediment microcosms amended with ammonium and nitrite, suggesting important roles for AOB in these mangrove ecosystems [16, 20]. However, archaeal *amoA* genes were more abundant than their bacterial counterparts in sediments from Florida covered with *A. germinans* [15], and in polluted Chinese mangrove sediments covered with *K. obovata* [19].

Based on the effects of mangrove species on microbial communities as described above, we hypothesized that the archaeal and bacterial ammonia-oxidizing communities in the root zones of *Avicennia* and *Rhizophora* species differ. We tested this hypothesis on soil samples collected from below *A. germinans* or *R. mangle* soil at the coast of Florida. Since former studies on ammonia-oxidizing archaea and bacteria were restricted to impounded *A. germinans* forests at the east coast with a restricted tidal regime [14, 15], we extended the sampling to tidal sites dominated by either *A. germinans* or *R. mangle*. We looked for differences in the ammonia-oxidizing communities by denaturing gradient gel electrophoresis (DGGE) of *amoA* genes and by MiSeq 16S rRNA gene-sequencing. We established also the abundance of AOA and AOB by means of quantitative amplification of *amoA* genes in the soils. Since the MiSeq 16S rRNA yielded also information on the diversity of the total archaeal and bacterial communities, we evaluated the diversity and composition of these communities as well. Furthermore, we explored the influence of physicochemical properties of the soils dominated by *A. germinans* and *R. mangle* on the total microbial communities based on 16S rRNA patterns.

## Materials and Methods

### Site Description and Soil Sampling

Soil sampling was performed at three locations at the coast of Florida. One sampling site was located on the western coast, at Port of the Islands (PI, 25° 54′ 48″ N, 81° 30′ 25″ W), Collier County. The other two sampling sites were on the western shores of a series of barrier islands located on the eastern coast of Florida; one of them at South Hutchinson Island (SHI, 27° 17′ 00″ N, 80° 13′ 00″ W), Martin County, and the other at North Hutchinson Island (NHI, 27° 33′ 09″ N, 80° 19′ 39″ W), in St. Lucie County. PI is a resort and marina at the Ten Thousand Islands National Islands Refuge, and an important habitat and refuge for aquatic life, including manatees [27]. SHI and NHI are part of the barrier islands that separate the Indian River Lagoon from the Atlantic Ocean. Some of the mangrove sites in this lagoon among which the sampling location at NHI, have been impounded to control noxious mosquitoes and midges [14]. Therefore, NHI has limited tidal exchange, whereas both SHI and PI are tidal locations. Further information on the sampling sites can be found in [28, 29]. At each location, four soil samples were collected from a site dominated by *A. germinans* and four samples from a site dominated by *R. mangle*, all near the roots of the trees, totalizing 24 samples (3 locations × 2 mangrove species × 4 replicates). The mutual distance between the samples at the site of one species was a few meters at the most. Upper 5-cm soil samples were collected using cores of 2-cm diameter, cooled and freeze-dried upon arrival in the laboratory for molecular analyses. At the sampling locations, additional upper 5-cm soil samples were collected for physical and chemical analyses of the soil [28]. Sample names were chosen according to the sampling site (PI, Port of the Islands; SHI, South Hutchinson Island; NHI, North Hutchinson Island), followed by the mangrove species (A, *A. germinans*; R, *R. mangle*), and the number of replicate (1–4).

### Characterization of Mangrove Soils

The concentrations of sulfur compounds and heavy metals in soil samples were determined by the Flemish Institute for Technological Research (VITO) in Belgium. Further metadata of these soil samples, including particle size, total organic carbon (TOC), pH, salinity, nitrate, and nitrite concentrations, have been previously described [28].

### Metagenomic DNA Extraction

Metagenomic DNA was purified from ca. 0.3 g of freeze-dried soil samples using the Maxwell® 16 Tissue DNA Purification Kit and Instrument (Promega Corporation, Madison, WI, USA), following the manufacturer’s instructions. DNA concentration and purity were measured with a NanoDrop 2000 Spectrophotometer (Thermo Fisher Scientific, Waltham, MA, USA). Further, DNA concentrations were determined using the QuantiFluor® dsDNA System (Promega Corporation, Madison, WI, USA), to corroborate the quantifications obtained using the NanoDrop.

### AOA and AOB Community Composition

The amplification of the *amoA* genes for DGGE was performed by nested PCR. The first PCR of the bacterial *amoA* genes was conducted using the *amoA*-1F/*amoA*-2R primer set [30] and the amplification conditions from Table 1, whereas that for the *amoA* genes of AOA was conducted using the Arch-amoAF/Arch-amoAR primers [11]. Then, a second PCR was performed using these PCR products as template DNA, the same primer sets as in the first round but with a GC clamp in the reverse primer, and the amplification conditions described in Table 1. PCR reactions contained 1× Green GoTaq® Flexi Buffer (Promega Corporation, Madison, WI, USA), 1 mM MgCl_2_, 0.4 mM dNTPs, 0.2 μM of each primer, 1.25 U GoTaq® G2 Hot Start Polymerase, 1–1.5 μl template DNA, and ultrapure water. Control reactions without DNA, as well as positive controls, were included in all runs. PCR products were analyzed by electrophoresis in 1.5% agarose gels to confirm the presence of bands of the specific size.

**Table 1:** Primers and programs used to amplify the *amoA* genes of ammonia-oxidizing archaea (AOA) and bacteria (AOB)

Primers	Target	Use	Amplification program	Reference
*amoA*-1F/*amoA*-2R	AOB β-Proteobacteria	qPCR, first PCR for DGGE	5 min at 95 °C, followed by 45 cycles of 20 s at 95 °C, 20 s at 59 °C, 20 s at 72 °C, and a final step of 15 s at 82 °C before fluorescence read	[30]
Arch-amoAF/AOA_amoA_175Brev	AOA	qPCR	5 min at 95 °C, followed by 45 cycles of 20 s at 95 °C, 30 s at 58 °C, 30 s at 72 °C, and a final step of 15 s at 82 °C before fluorescence read	[11, 31]
Arch-amoAF/Arch-amoAR	AOA	First PCR for DGGE	10 min at 95 °C, followed by 45 cycles of 15 s at 95 °C, 45 s at 56 °C and 45 s at 72 °C	[11]
*amoA*-1F/*amoA*-2R-GC clamp^a^	AOB β-Proteobacteria	Second PCR for DGGE	3 min at 95 °C, followed by 10 cycles of 30 s at 95 °C, 45 s at 60 °C with a 0.5 °C decrease per cycle and 60 s at 72 °C, followed by 30 cycles of 30 s at 94 °C, 45 s at 55 °C and 60 s at 72 °C, and a final elongation step of 10 min at 72 °C [32]	[30]
Arch-amoAF/Arch-amoAR-GC clamp^a^	AOA	Second PCR for DGGE	5 min at 95 °C, followed by 30 cycles of 45 s at 95 °C, 45 s at 54 °C with a 0.2 °C increment per cycle, and 60 s at 72 °C, and a final elongation step of 10 min at 72 °C	[11]

^a^GC clamp: 5′ CGCCCGCCGCGCGCGGCGGGCGGGGCGGGGGCACGGGGGG 3′ [33]

PCR products were loaded in 6% polyacrylamide gels with 20–55% and 35–60% denaturing gradients for archaeal and bacterial genes, respectively (where 100% denaturant was 7 M urea and 40% formamide in 0.5× TAE buffer). Electrophoresis was carried in a PROTEAN® II xi Cell (Bio-Rad Laboratories, Hercules, CA, USA) using 0.5× TAE buffer. Gels were run at 60 °C, during 17 h at 100 V (AOA) or 12 h at 80 V (AOB), and then stained with ethidium bromide and visualized in a transilluminator. The banding pattern of DGGE was compared among samples using the Image master 1D Database Software.

### Quantification of amoA Genes from AOA and AOB

Real-time PCR assays to quantify the bacterial and archaeal *amoA* genes in soil samples were performed in a Rotor-Gene® Q thermocycler (QIAGEN, Hilden, Germany), using the primers and amplification programs described in Table 1. All reactions contained 1× SYBR® Green Master Mix (Bio-Rad Laboratories, Hercules, CA, USA), 5 μg ml^−1^ BSA, 250 nM of each primer, 1:40–1:4 dilution template DNA (depending on the presence of inhibitors and the abundance of the target gene in each DNA sample), and ultrapure water. Control reactions, where template DNA was replaced by ultrapure water, were included in all runs. Melting curves were run at the end of the program to verify the specificity of the amplified products. Standard curves were constructed by performing 1:10 serial dilutions of *amoA* genes amplified from uncultured AOB and AOA, in the ranges of 5–10^7^ (*r* ^2^ > 0.98) and 10^2^–10^9^ (*r* ^2^ > 0.99) for bacterial and archaeal genes, respectively.

### Microbial Community Structure and Diversity

Bacterial and archaeal 16S rRNA genes of the 24 soil DNA samples were amplified and sequenced at BGI (Copenhagen, Denmark). The 515F/806R primer set [34] was used to amplify the V4 hypervariable region of 16S rRNA genes from both Bacteria and Archaea. Paired-end sequencing was performed using an Illumina MiSeq Sequencing platform. Sequences were processed using the software QIIME v 1.9.1 [35]. Briefly, forward and reverse sequences were joined, demultiplexed, and filtered according to quality scores (< 25 were removed), chimeric sequences, and read length (< 200 bp were removed). Five of the 24 DNA samples (NHI-A2, NHI-R3, PI-A1, PI-R1, and PI-R2) produced less than 500 reads, and therefore were discarded from further analyses. After filtering steps, we had a total of 117,649 reads from 19 samples (minimum: 966, maximum: 14,506 reads per sample). Sequences were aligned using the SILVA database v 128 as template [36]. Operational taxonomic units (OTUs) were picked at 97% sequence similarity using UCLUST, and taxonomy was assigned using the SILVA database v 128 [36].

The Good’s coverage index was calculated for each soil sample, as a measure of the depth of sequencing effort. Alpha diversity metrics (total observed OTUs, Simpson evenness (1/D/S), and the Shannon index (H′) as richness, evenness, and diversity estimators, respectively) were calculated based on a subsampled OTU table of 950 sequences (to fit the smallest library size obtained), since these metrics tend to vary with library size. All these metrics were calculated using QIIME v 1.9.1 [35]. The Bray-Curtis beta diversity index was calculated using PRIMER-E v 7 [37] to measure similarity between soil samples. Before Bray-Curtis index calculation, relative abundances of each OTU in a soil sample were calculated by dividing OTU abundance by the total amount of sequences in that sample; then OTU relative abundances were square root-transformed to down-weight the importance of very abundant species [38]. The Bray-Curtis similarity matrix was further used to evaluate similarities in microbial community structure of soils covered by *A. germinans* and *R. mangle*.

### Statistical and Ordination Analyses

Differences in community alpha diversity estimators (total observed OTUs, Simpson evenness (1/D/S), and the Shannon (H′) indices) in soils underneath *A. germinans* and *R. mangle* were tested with a nonparametric Kruskal Wallis test [39], as pairwise comparisons within each sampling site. Nonmetric multidimensional scaling (NMDS) and cluster analyses were performed to evaluate similarities in soil microbial community structures, based on a Bray-Curtis similarity matrix of (square root-transformed) 16S rRNA OTU relative abundances. Further, a Permutational Multivariate Analysis of Variance (PERMANOVA) [40], based on the same Bray-Curtis similarity matrix, and an analysis of similarities (ANOSIM) were performed to test for significant differences in overall microbial community structures. In soil samples with different community structures due to mangrove species (detected by cluster and ANOSIM analyses), a Similarity Percentages (SIMPER) analysis of taxonomically classified sequences (phylotype-based approach) was performed to detect the taxa with larger contributions to those differences, using PRIMER-E v 7 [37]. The similarity in AOA and AOB community composition (obtained by PCR-DGGE of *amoA* genes) was evaluated by a cluster analysis of the banding pattern of DGGE based on the Sorensen similarity index. Further, similarities in the composition of AOB of the family Nitrosomonadaceae (obtained by MiSeq analysis) were evaluated by cluster and ANOSIM analyses. All multivariate analyses were performed using PRIMER-E v 7 [37].

Differences in the abundance of (log-transformed) *amoA* genes from AOB and AOA in response to the mangrove species within each location were tested using one-way ANOVA (previous testing the assumptions of normality and homoscedasticity). The similarity in soil environmental factors of samples underneath different mangrove species and from different sampling locations was explored by principal component analysis (PCA). Before PCA, environmental variables measured as concentrations were log-transformed and all environmental variables were standardized to zero mean and standard deviation of one, to avoid different measure units in the multivariate analysis.

### Accession Number

Sequences of bacterial and archaeal 16S rRNA genes were deposited in the European Nucleotide Archive (ENA) under the accession number PRJEB22120.

## Results

### Abundance of AOA and AOB

**Fig. 1:**
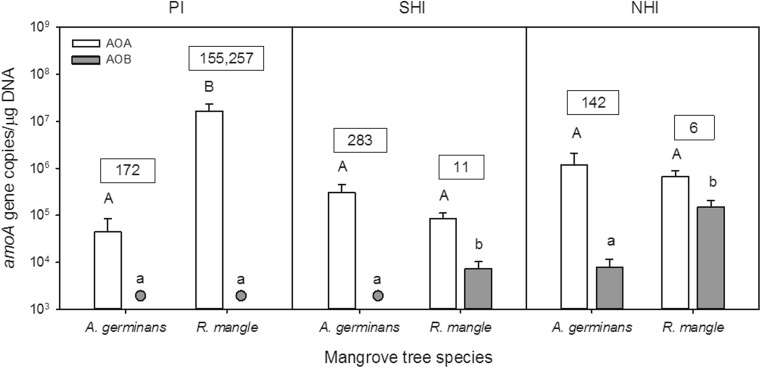
Abundance of archaeal (AOA) and bacterial (AOB) *amoA* gene copies in samples of soil covered with *Avicennia germinans* and *Rhizophora mangle*. Error bars represent the standard error of the data (*n* = 4). Abundances of bacterial gene copies below the quantification limit of the assay (1200 gene copies per μg DNA) are represented by gray circles. The ratio of archaeal to bacterial *amoA* genes (AOA/AOB) is indicated in the boxes on top of the bars. Within each location, significant differences in the abundance of *amoA* genes in soils covered with different mangrove species are indicated with different letters above the bars (uppercase letters, comparisons between archaeal *amoA* genes; lowercase letters, comparisons between bacterial *amoA* genes)

The effect of mangrove species on the abundance of AOA and AOB was studied by quantifying the archaeal and bacterial *amoA* genes in samples of soils covered with *A. germinans* and *R. mangle*. The abundance of *amoA* genes from AOA ranged from 4.4 ± 8.0 × 10^4^ to 1.7 ± 1.3 × 10^7^ gene copies per microgram DNA (Fig. 1). In contrast, bacterial *amoA* genes ranged from abundances below the quantification limit of the assay (1200 gene copies per μg DNA) to 1.5 ± 1.2 × 10^5^ gene copies per μg DNA and were outnumbered by archaeal genes by ratios of 6 to 1.6 × 10^5^ (Fig. 1). We observed significant effects of mangrove species on AOA gene abundance only in soil samples from PI, where soils covered with *R. mangle* had almost three orders of magnitude higher gene abundances than soils covered with *A. germinans* (*p* < 0.005). In soil samples from the two remaining locations, AOB genes were significantly more abundant in soils covered with *R. mangle* than in those underneath *A. germinans* (*p*_NHI_ = 0.01, *p*_SHI_ = 0.03).

### Composition of AOB and AOA Communities

Our first approach to test the effect of tree species on the community composition of ammonia oxidizers was performed by PCR-DGGE based on *amoA* genes. With respect to the archaeal *amoA* gene, samples of soils covered with *A. germinans* separated completely from samples of soil covered by *R. mangle*, except for only two samples from NHI (i.e., NHI-A1 and NHI-A4, Fig. S1). In contrast, cluster analyses of bacterial *amoA* genes showed different results at each location. Samples of soils from SHI covered with *R. mangle* grouped together with similarity values higher than 70%, but those covered with *A. germinans* did not group altogether. Except for one of the *Avicennia* locations that showed low amplification product (i.e., NHI-A4), soil samples from NHI showed very similar banding patterns (higher than 80% similar), independently of the type of mangrove covering the soil. Lastly, cluster analysis of soil samples from PI could not be performed, due to very weak bands or lack of amplification of the *amoA* genes from AOB in most of the samples (probably due to low gene abundance in samples from this site).

We further analyzed the effect of tree species on ammonia oxidizer community composition by MiSeq analysis of 16S rRNA genes in soil samples. Within the bacteria, sequences assigned to the genus *Nitrosomonas* were detected in all samples, at abundances between 0.3 and 0.9% of the total number of sequences. The genera *Nitrosospira* and *Nitrosococcus* were not detected, although it is possible that sequences within the families Nitrosomonadaceae and Chromatiaceae that were classified as uncultured or could not be classified at the genus level, were related to these genera, respectively. Cluster and ANOSIM analyses based on sequences classified as Nitrosomonadaceae completely separated soil samples covered by *A. germinans* from those covered by *R. mangle* within locations SHI and PI, but not within NHI (cluster analysis in Fig. S2, and ANOSIM global *R* = 0.568 (*p* = 0.001), *R*_SHI_ = 0.792, *R*_PI_ = 1, *R*_NHI_ = 0). In contrast to AOB, known AOA genera were not detected in soil samples. This is probably a consequence of the low abundance of cultured Archaea in the 16S rRNA databases, since 34 to 88% of the archaeal sequences were assigned to uncultured microorganisms at the genus level.

### Bacterial and Archaeal Community Structure and Diversity

A total of 117,649 reads were obtained from 19 soil samples, with an average of 7740 sequences per sample. Five samples (NHI-A2, NHI-R3, PI-A1, PI-R1, and PI-R2) were discarded from further analyses because they produced less than 500 reads. The Good’s coverage index was on average higher than 90%, suggesting that sequencing effort was good in most samples (Table 2). In soil samples from PI, OTU richness, the Simpson evenness, and Shannon diversity indices were significantly higher in soils underneath *R. mangle* than in soils beneath *A. germinans* (*p* < 0.01, Table 2). Soil samples from the other locations did not show significant differences in richness, diversity, and evenness between both mangrove species.

**Table 2:** Soil microbial alpha diversity estimators based on OTUs at 97% similarity (average ± standard error). Significant differences in pairwise comparison within the same sampling site are indicated with asterisks (** *p* < 0.01)

Sample	Good’s coverage^a^	Observed OTUs^b^	Simpson evenness (1/D/S)^b^	Shannon (H′)^b^
PI-A	0.94 ± 0.03	312 ± 30.5 ^**^	0.035 ± 0.013 ^**^	5.98 ± 0.58 ^**^
PI-R	0.94 ± 0.03	449 ± 25.2 ^**^	0.248 ± 0.046 ^**^	8.00 ± 0.20 ^**^
SHI-A	0.95 ± 0.01	368 ± 26.0	0.141 ± 0.073	7.14 ± 0.36
SHI-R	0.89 ± 0.02	388 ± 25.3	0.171 ± 0.077	7.32 ± 0.45
NHI-A	0.92 ± 0.04	384 ± 7.7	0.219 ± 0.048	7.64 ± 0.14
NHI-R	0.84 ± 0.06	379 ± 47.2	0.162 ± 0.079	7.08 ± 0.81

^a^Calculation based on all the sequences of each sample

^b^Calculation based on a subsampled OTU table of 950 sequences, to fit the size of the smallest library

**Fig. 2:**
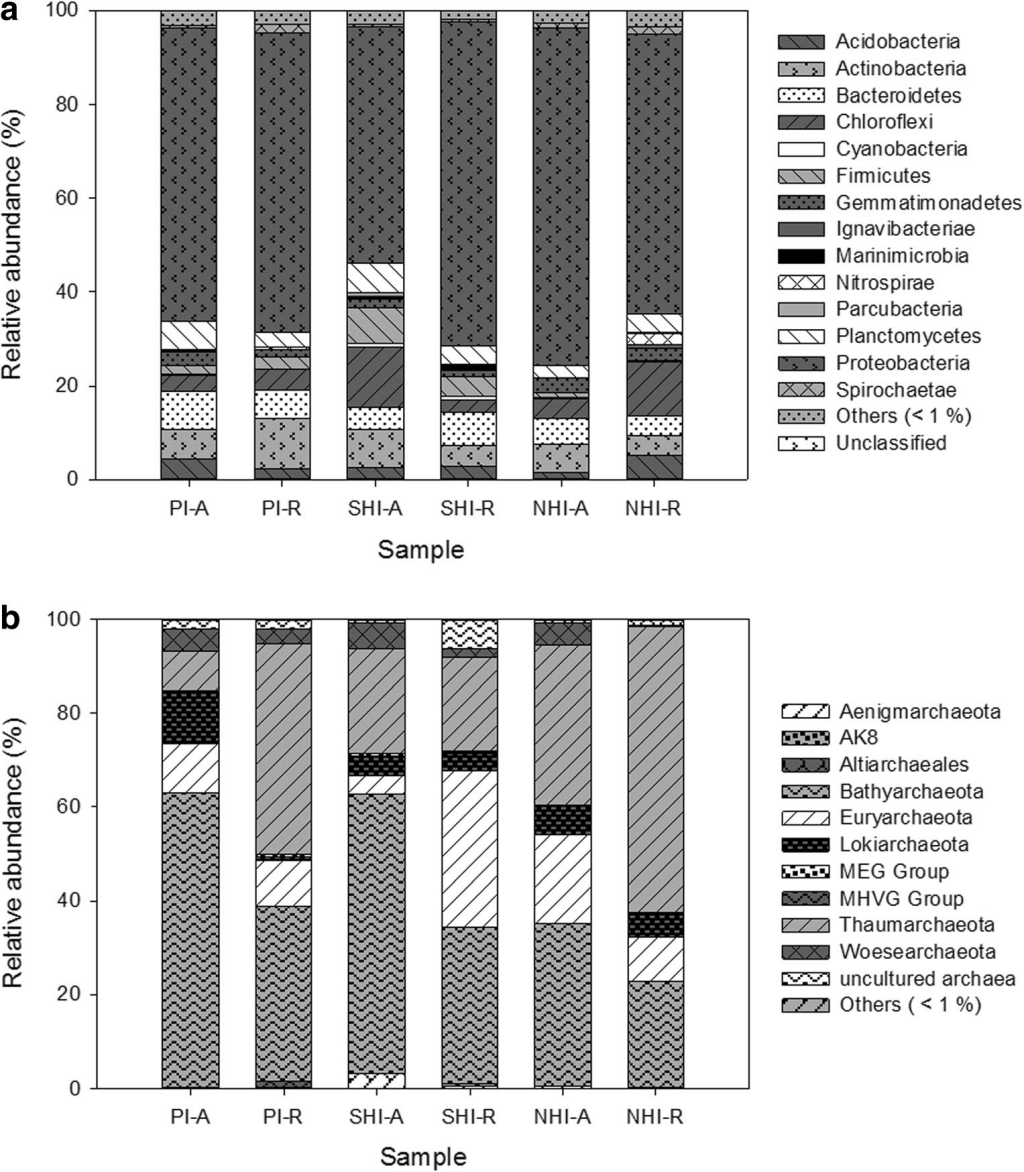
Relative abundance of bacterial (**a**) and archaeal (**b**) phyla in forest soils dominated by *Avicennia germinans* (PI-A, SHI-A and NHI-A) or *Rhizophora mangle* (PI-R, SHI-R and NHI-R)

The grouping of sequences at a 97% similarity threshold resulted in 3925 OTUs, of which 153 were classified as Archaea, 3593 as Bacteria, and 179 were unclassified. Bacteria represented 83.4 to 98.7% of the 16S rRNA sequences from soil samples, and were on average 50-fold more abundant than Archaea, that represented 0.7 to 15.9% of the total soil sequences. The most representative bacterial phyla were Proteobacteria (relative abundance 45.1–82.6%), Actinobacteria (3.0–11.5%), Bacteroidetes (1.2–19.6%), Chloroflexi (1.3–25.1%), Planctomycetes (0.8–8.0%), Acidobacteria (1.4–6.1%), Gemmatimonadetes (0.8–5.2%), and Firmicutes (0.4–10.0%) (Fig. 2a). In addition, 36 bacterial phyla were detected at lower abundances (< 1% average among all samples). Regarding the archaeal community, the dominant phylum was Bathyarchaeota (19.5–80.2%), followed by Thaumarchaeota (2.4–73.0%), Euryarchaeota (1.4–41.9%), Woesearchaeota (0–17.0%), and Lokiarchaeota (0–14.6%). The remaining sequences were either at low abundance (< 1% average among all samples), or classified as uncultured Archaea (Fig. 2b).

**Fig. 3:**
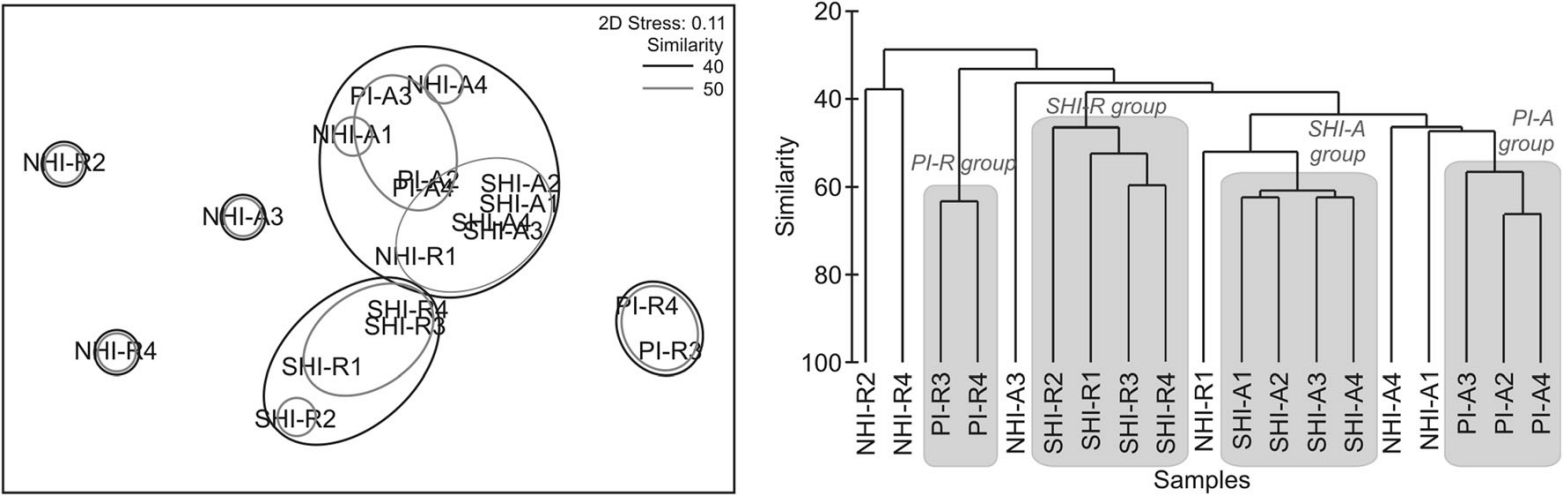
NMDS (left panel) and cluster (right panel) analyses based on Bray-Curtis similarities of microbial communities from soil samples dominated by *Avicennia germinans* (PI-A, SHI-A and NHI-A) or *Rhizophora mangle* (PI-R, SHI-R and NHI-R)

A PERMANOVA test revealed that the community structures in soil samples from different locations and mangrove cover differed significantly (Pseudo-*F* = 3.15, *p* = 0.001). Further, an analysis of OTUs at 97% similarity showed that soil samples from PI and SHI underneath a single mangrove cover had similar microbial community structures, as revealed by cluster and NMDS analyses (Fig. 3). In contrast, soil samples from NHI did not group together, although two soil samples from this location underneath *A. germinans* (NHI-A1 and NHI-A4) grouped with soil samples from another location but with similar vegetation cover (PI-A group, Fig. 3). An analysis of similarities (ANOSIM) confirmed the differences in community structures of soil samples from different locations and mangrove species (global *R* = 0.754, *p* < 0.001), and pairwise comparisons detected differences in community structures due to mangrove species cover in soil samples from SHI (*R* = 1) and PI (*R* = 1), but not in samples from NHI (*R* = 0.37). Further, a SIMPER analysis was performed to detect the taxa that mostly contributed to differences between mangrove species in SHI and PI. This analysis showed that bacterial genera with larger contributions to differences in community structure due to mangrove species were *Marinifilum* and *Tenacibaculum* belonging to the phylum Bacteroidetes, and *Pseudolabrys*, *Arcobacter*, *Sulfurimonas*, *Sulfurovum*, *Thioalkalispira*, and *Sedimenticola* of the phylum Proteobacteria in SHI; and uncultured Anaerolineaceae of the phylum Chloroflexi and *Sulfurovum* and *Thioalkalispira* of the phylum Proteobacteria in PI (Table S1). Archaeal groups with larger contributions to differences between mangrove species were uncultured Bathyarchaeota, *Methanococcoides*, uncultured and unclassified archaea belonging to the Marine Benthic Group D (DHVEG-1) of the phylum Euryarchaeota, and uncultured archaea of the Marine group I of the phylum Thaumarchaeota in SHI; and uncultured Bathyarchaeota, uncultured Lokiarchaeota, and uncultured and unclassified archaea of the phylum Thaumarchaeota in PI (Table S2).

### Soil Physicochemical Properties

Physicochemical properties of soil samples are reported in Table S3. By pairwise comparison per sampling location, *A. germinans* soil samples had higher concentrations of some nutrients (i.e., sulfur, calcium, and magnesium), although *R. mangle* soil samples had higher concentrations of iron. In addition, samples of soils covered with *A. germinans* were slightly more saline (with higher concentrations of sodium) than those covered with *R. mangle* trees. Salinity was usually above seawater levels, pH values were generally neutral (except for SHI-A that was slightly more acidic) and TOC was lower in soil samples from NHI than in those from PI and SHI, as previously reported [28].

We used PCA to explore the differences in physicochemical properties between soil samples (Fig. S3). The first principal component (PC1) explained 58.4% of the variation and was determined by several variables with relatively equal importance (nickel, chromium, zinc, magnesium, cobalt, sodium, TOC, particle size, potassium, copper, salinity, iron, sulfate, phosphorus, aluminum, and sulfur). This PC completely separated samples from tidal locations (PI-A, PI-R, SHI-A, and SHI-R, to the left of the graph) from samples retrieved at non-tidal locations (NHI-A and NHI-R, to the right). The second principal component (PC2) explained 19.9% of the variation, which led to a total explanation of the variation of 78.3%. Manganese, calcium, and iron were the variables that mostly contributed to the determination of PC2.

## Discussion

In this study, we aimed to elucidate the effect of two species of globally distributed mangrove genera (*Avicennia* and *Rhizophora*) on ammonia-oxidizing microbial communities in the soil. *Avicennia* and *Rhizophora* differ in their resource acquisition and survival strategies, stress tolerance, root adaptations, and tissue chemistry, and are therefore adapted to different zones in a tidal gradient. We hypothesized that species of ammonia-oxidizing archaea and bacteria, like mangrove species, are adapted to specific tidal zones and hence indirectly coupled with specific mangrove species. This hypothesis was confirmed for the community composition of the ammonia oxidizers at the two tidal locations (PI and SHI), but did not apply to the non-tidal location of NHI, reflecting the results previously obtained with samples from the same mangrove locations when the sulfate-reducing community was studied [41]. Moreover, the difference observed for community structures of AOA and AOB was reflected by the difference in 16S rRNA gene community structures for the whole archaeal and bacterial communities, i.e., only differences at the tidal locations. The presence of differences in microbial communities only at the tidal locations and not at the non-tidal location demonstrates that the microbial community structure is primarily governed by the presence of a tidal regime, and not by the dominant mangrove species. The fact that no differences were observed between the soil samples collected from beneath *A. germinans* and *R. mangle* at the non-tidal location implies that a differential effect on the soil microbial community triggered by these species is rather limited.

Two different tools were used to test for differences in ammonia-oxidizing communities in soils underneath *A. germinans* or *R. mangle*. On the one hand, the DGGE analysis based on *amoA* genes detected differences in AOA communities associated with different mangrove species, but was not able to detect differences in AOB, probably because this fingerprinting technique does not have enough resolution power to detect differences in *amoA* genes of AOB present at low abundance. On the other hand, cluster and ANOSIM analyses of Nitrosomonadaceae sequences obtained by MiSeq completely separated soil samples covered by *A. germinans* from those covered by *R. mangle* at both tidal locations, although this technique was not able to detect differences in AOA composition. This may be a consequence of the low abundance of cultured representatives of archaea in 16S rRNA databases used to classify sequences, since 34 to 88% of the archaeal sequences in this study were assigned to uncultured microorganisms at the genus level. In accordance, it has been reported that 14 of the 20 existing archaeal phyla have no cultured representatives, and that among the phyla with cultured representatives, only 1.7% of the sequences came from cultures (the remaining sequences coming from culture-independent approaches) [42]. Overall, these two approaches revealed complementary information about the composition of AOB and AOA in these mangrove soils.

In comparison to soil beneath *A. germinans*, soils associated with *R. mangle* had a higher abundance of AOA genes at PI, and of AOB genes at NHI and SHI. Hence, the effect of mangrove species on the sizes of the AOA and AOB communities were different for the east and west coast of Florida, whereas tide had apparently, no effect on the relative sizes of both ammonia-oxidizing communities at the east coast. Since the effects on the relative community sizes of AOA and AOB are site-specific and not mangrove species-specific, the responsible mechanism behind the observed differences must also be site-specific. Because total nutrient concentrations were the lowest in *R. mangle* soil samples from PI, the high relative abundance of AOA in these soils could be related to the capacity of these ammonia oxidizers to thrive and outcompete AOB in oligotrophic environments [43, 44]. Further studies should be directed to identify site-specific soil and environmental characteristics that could be influencing the abundances of AOA and AOB communities.

Studies of the relationship between AOB and AOA abundances in mangrove ecosystems have hitherto shown contrasting results. Mangroves from the Mai Po Marshes Nature Reserve (China) dominated by *K. obovata* showed higher abundances of bacterial than archaeal *amoA* genes (AOB/AOA gene ratio ca. 1 to 12) and suggested that AOB might play a more important role in mangrove sediments than AOA [20]. A seasonal study under vegetated and non-vegetated sediments in the same Nature Reserve showed slightly higher abundances of AOB than AOA genes, with AOB/AOA gene ratio ranging from 0.7 to 3.6 [17]. Further studies performed in Chinese mangrove ecosystems showed that not only bacterial *amoA* genes but also gene transcripts were more abundant than those of archaea [16, 18]. In contrast, other studies found higher abundances of archaeal than bacterial ammonia oxidizers in mangrove sediments. In polluted mangrove sediments from China, archaeal *amoA* genes were 1.8 to 6.3 times more abundant than their bacterial counterpart, and the AOA/AOB ratio was correlated with ammonium concentration [19]. Besides these studies in Southeast Asia, a previous study in sediments covered with *A. germinans* at the coast of Florida showed a dominance of archaeal over bacterial *amoA* genes independently of sampling year, impoundment sampled or mangrove vegetation cover type, with AOA/AOB gene ratios varying largely between 0.9 and 6.5 × 10^4^ [15]. Our results agreed with these latter results in that AOA genes were up to several orders of magnitude more abundant than those of AOB and further support a dominance of AOA over AOB in mangrove sediments from this region. These results may suggest that AOB could be more sensitive and more easily influenced by environmental factors as previously observed by Cao and colleagues [19], whereas AOA are probably less sensitive and therefore became dominant in this environment. Alternatively AOA may be more resistant to decay than AOB. Measuring *amoA* transcripts might be a way to resolve this problem, but then it should be kept in mind that inactive AOA may preserve their *amoA* transcripts for a longer period than inactive AOB [45].

In general, the effect of mangrove species on total soil bacterial and archaeal community compositions has hardly been characterized. Bacterial communities in the rhizospheres of *Avicennia schaueriana* and *Laguncularia racemosa* and archaeal communities in the rhizospheres of *R. mangle* and *L. racemosa* from Brazil showed some differences in their composition, although the main variation was between the rhizosphere of either mangrove tree and the bulk sediment communities [24, 25]. Archaeal communities were also studied in mangroves from China, and differences were found between communities associated to *K. candel* or *Bruguiera gymnoihiza* and those associated to *L. racemosa* or *Sonneratia apetala* mangroves [22].

At PI, differences in the bacterial component of the microbial community were mostly attributed to increased abundances of *Sulfurovum* and *Thioalkalispira* in soils covered by *A. germinans* and *R. mangle*, respectively. These bacteria are both halophilic, facultative anaerobes or microaerophiles, and sulfur or thiosulfate oxidizers [46, 47] that may play similar roles in the cycling of sulfur in both soil types, but are specifically enriched by unknown habitat-specific characteristics. Soils underneath *A. germinans* also had higher abundances of uncultured Lokiarchaeota and Bathyarchaeota, and lower abundance of Thaumarchaeota than the soils covered by *R. mangle*. Bathyarchaeota and Thaumarchaeota, which together represented 43.3 to 94.3% of the archaeal sequences in these mangroves, have been detected in diverse habitats and often at high abundances, suggesting a high ability to adapt to different environmental conditions, probably as a result of versatile metabolic pathways [48, 49]. Regarding the phylum Lokiarchaeota, it seems to be abundant and widely distributed in deep sediments, but also in mangrove soils [50]. Although so far little is known about this group since archaea of this phylum have never been isolated or enriched [51], they could play a role in anaerobic biogeochemical processes such as sulfate reduction or methane oxidation [52]. In agreement with this, mangrove soils tend to be dominated by anaerobic methanogens and sulfate-reducing bacteria [24, 25, 53].

Several microorganisms contributed to the differences in community structures of soils covered by *A. germinans* and *R. mangle* in SHI. *R. mangle*-covered soils had a higher abundance of strictly or facultative anaerobic bacteria of the genera *Sedimenticola*, *Thioalkalispira*, and *Marinifilum*, and a lower abundance of *Pseudolabrys* than *A. germinans*-covered soils. Hardly anything is known about the ecology of *Pseudolabrys* [54]; however, its higher abundance in soils underneath *A. germinans* than *R. mangle* could be related to its aerobic metabolism and the capacity of *Avicennia* species to maintain more oxidized soil conditions than *Rhizophora* [55]. However, also anaerobic and microaerophilic bacteria (*Sulfurovum*, *Arcobacter*, *Tenacibaculum*) dominated *A. germinans*-covered soils in SHI, showing the dynamic character of tidal mangrove soils with respect to oxygen availability. Except for *Sulfurovum*, these genera were present at high abundance in only one of the samples and nearly absent in all the others; therefore, we cannot generalize that their abundance is related to a particular mangrove species. Uncultured and unclassified archaea belonging to the Marine Benthic Group D were more abundant underneath *R. mangle* than *A. germinans* soils. This lineage belongs to the class Thermoplasmata, a group of facultatively anaerobic, thermoacidophilic archaea capable of respiring sulfur [56]. In spite of their acidophilic metabolism, Thermoplasmata have been previously detected at very high abundances (70.4% of the archaeal sequences) in mangrove soils of neutral pH [57]. In accordance, we found a high abundance of these archaea in soil samples of neutral pH and with high concentrations of sulfur. Therefore, it could be possible that they were growing in acidic microenvironments within these mangrove soils where elemental sulfur is reduced to H_2_S.

In contrast to SHI and PI, the non-tidal location NHI showed no differences in microbial communities from *A. germinans-* and *R. mangle*-covered soils. This result reflects the results obtained with samples from the same mangrove locations when sulfate reduction characteristics were studied [41]. At the tidal locations PI and SHI, steady state sulfate reduction rates, and *dsrB* gene copy numbers were higher at the *A. germinans* than at the *R. mangle* stands, although not significantly for the numbers at PI. At the non-tidal NHI location, results were mixed with respect to the sulfate reduction traits. Impounding can cause limited tidal exchange, changes in water quality [58], in salinity due to hydrological alterations and evapotranspiration [59], in sediment chemistry, nutrient dynamics and redox conditions [29, 60], in plant and fish communities [61], and also in soil microbial communities [41]. In this study, the limited tidal exchange at the impounded location of NHI might have had a stronger effect on microbial communities than vegetation, since the microbial communities had very variable compositions independently of the plant cover, which precluded us from finding a characteristic microbiome associated with each mangrove species at this location. In accordance, the PCA based on soil physicochemical properties from our study completely separated soil samples retrieved at tidal stations from those collected at the non-tidal location, independently of the vegetation cover, suggesting that tide instead of mangrove species had the largest effect on soil physicochemical properties. Nonetheless, this represents a first exploratory description of mangrove soil physicochemical properties, and further studies should be performed to establish their effect on the soil microbial communities in tidal and non-tidal locations.

Overall, this is the first study that compares the effect of two different species of globally distributed mangrove genera (*Rhizophora* and *Avicennia*) on soil bacterial and archaeal communities, and on the functional group of ammonia oxidizers. Mangrove trees influenced both the composition of AOA and AOB and that of the overall soil microbial communities, but only in locations exposed to tides. The absence of tidal exchange in the impounded location might have had a stronger effect on community compositions than the vegetation cover. In addition, we showed that the relative abundances of archaeal and bacterial *amoA* genes is site-specific and not mangrove tree-specific. However, independently of site and mangrove species, AOA outnumbered AOB at all sampling locations, suggesting a dominance of archaeal over bacterial ammonia oxidizers in mangrove sediments from this region.

## Electronic supplementary material

ESM 1 (PDF 911 kb)

## References

[1] Walters BB, Rönnbäck P, Kovacs JM (2008). Ethnobiology, socio-economics and management of mangrove forests: a review. Aquat Bot.

[2] Smith III TJ (1992) Forest structure. In: Robertson AI, Alongi DM (eds) Trop. mangrove Ecosyst. Washington D.C., pp 101–136

[3] Mckee KL (1993). Soil physicochemical patterns and mangrove species distribution - reciprocal effects?. J Ecol.

[4] FAO (2007) The world’s mangroves 1980–2005. Rome

[5] McKee KL (1995). Interspecific variation in growth, biomass partitioning, and defensive characteristics of neotropical mangrove seedlings: response to light and nutrient availability. Am J Bot.

[6] Alongi DM, Tirendi F, Clough BF (2000). Below-ground decomposition of organic matter in forests of the mangroves *Rhizophora stylosa* and *Avicennia marina* along the arid coast of Western Australia. Aquat Bot.

[7] Rao RG, Woitchik AF, Goeyens L (1994). Carbon, nitrogen contents and stable carbon isotope abundance in mangrove leaves from an east African coastal lagoon (Kenya). Aquat Bot.

[8] Keuskamp JA (2014) Decomposition and soil carbon sequestration in mangrove ecosystems. PhD Thesis. Utrecht University

[9] Schleper C, Nicol GW (2010). Ammonia-Oxidising Archaea - physiology, ecology and evolution. Adv Microb Physiol.

[10] Kowalchuk GA, Stephen JR (2001). Ammonia-oxidizing bacteria: A model for molecular microbial ecology. Annu Rev Microbiol.

[11] Francis CA, Roberts KJ, Beman JM (2005). Ubiquity and diversity of ammonia-oxidizing archaea in water columns and sediments of the ocean. Proc Natl Acad Sci USA.

[12] Junier P, Molina V, Dorador C (2010). Phylogenetic and functional marker genes to study ammonia-oxidizing microorganisms (AOM) in the environment. Appl Microbiol Biotechnol.

[13] Prosser JI, Nicol GW (2012). Archaeal and bacterial ammonia-oxidisers in soil: the quest for niche specialisation and differentiation. Trends Microbiol.

[14] Laanbroek HJ, Keijzer RM, Verhoeven JTA, Whigham DF (2012). The distribution of ammonia-oxidizing betaproteobacteria in stands of Black mangroves (*Avicennia germinans*). Front Microbiol.

[15] Laanbroek HJ, Keijzer RM, Verhoeven JTA, Whigham DF (2013). Changes in community composition of ammonia-oxidizing betaproteobacteria from stands of Black mangrove (*Avicennia germinans*) in response to ammonia enrichment and more oxic conditions. Front Microbiol.

[16] Li M, Gu JD (2013). Community structure and transcript responses of anammox bacteria, AOA, and AOB in mangrove sediment microcosms amended with ammonium and nitrite. Appl Microbiol Biotechnol.

[17] Wang YF, Feng YY, Ma X, Gu JD (2013). Seasonal dynamics of ammonia/ammonium-oxidizing prokaryotes in oxic and anoxic wetland sediments of subtropical coastal mangrove. Appl Microbiol Biotechnol.

[18] Wang H, Su J, Zheng T, Yang X (2015). Insights into the role of plant on ammonia-oxidizing bacteria and archaea in the mangrove ecosystem. J Soils Sediments.

[19] Cao H, Li M, Hong Y, Gu JD (2011). Diversity and abundance of ammonia-oxidizing archaea and bacteria in polluted mangrove sediment. Syst Appl Microbiol.

[20] Li M, Cao H, Hong Y, Gu JD (2011). Spatial distribution and abundances of ammonia-oxidizing archaea (AOA) and ammonia-oxidizing bacteria (AOB) in mangrove sediments. Appl Microbiol Biotechnol.

[21] Wickramasinghe S, Borin M, Kotagama SW (2009). Multi-functional pollution mitigation in a rehabilitated mangrove conservation area. Ecol Eng.

[22] Li W, Guan W, Chen H (2016). Archaeal communities in the sediments of different mangrove stands at Dongzhaigang, China. J Soils Sediments.

[23] Yang Q, Lei AP, Li FL (2014). Structure and function of soil microbial community in artificially planted *Sonneratia apetala* and *S. caseolaris* forests at different stand ages in Shenzhen Bay, China. Mar Pollut Bull.

[24] Gomes NCM, Cleary DFR, Pires ACC (2014). Assessing variation in bacterial composition between the rhizospheres of two mangrove tree species. Estuar Coast Shelf Sci.

[25] Pires ACC, Cleary DFR, Almeida A (2012). Denaturing gradient gel electrophoresis and barcoded pyrosequencing reveal unprecedented archaeal diversity in mangrove sediment and rhizosphere samples. Appl Environ Microbiol.

[26] Prescott CE, Grayston SJ (2013). Tree species influence on microbial communities in litter and soil: current knowledge and research needs. For Ecol Manag.

[27] Swain ED, Decker JD (2009) Development, testing, and application of a coupled hydrodynamic surface-water/groundwater model (FTLOADDS) with heat and salinity transport in the Ten Thousand Islands/Picayune Strand Restoration Project area, Florida. Reston, Virginia, USA

[28] Balk M, Laverman AM, Keuskamp JA, Laanbroek HJ (2015). Nitrate ammonification in mangrove soils: a hidden source of nitrite?. Front Microbiol.

[29] Verhoeven JTA, Laanbroek HJ, Rains MC, Whigham DF (2014). Effects of increased summer flooding on nitrogen dynamics in impounded mangroves. J Environ Manag.

[30] Rotthauwe JH, Witzel KP, Liesack W (1997). The ammonia monooxygenase structural gene *amoA* as a functional marker: molecular fine-scale analysis of natural ammonia-oxidizing populations. Appl Env Microbiol.

[31] Laanbroek HJ, Veenhuizen PTM, Keijzer RM, Hefting MM (2017) Numerical relationships between archaeal and bacterial *amoA* genes vary by Icelandic Andosol classes. Microb Ecol. 10.1007/s00248-017-1032-9 10.1007/s00248-017-1032-9 PMC5742608 28707145

[32] Chen X, Zhang LM, Shen JP (2010). Soil type determines the abundance and community structure of ammonia-oxidizing bacteria and archaea in flooded paddy soils. J Soils Sediments.

[33] Muyzer G, De Waal EC, Uitterlinden AG (1993). Profiling of complex microbial populations by denaturing gradient gel electrophoresis analysis of polymerase chain reaction-amplified genes coding for 16S rRNA. Appl Environ Microbiol.

[34] Caporaso JG, Lauber CL, Walters WA (2011). Global patterns of 16S rRNA diversity at a depth of millions of sequences per sample. Proc Natl Acad Sci USA.

[35] Caporaso JG, Kuczynski J, Stombaugh J (2010). QIIME allows analysis of high-throughput community sequencing data. Nat Methods.

[36] Quast C, Pruesse E, Yilmaz P (2013). The SILVA ribosomal RNA gene database project: improved data processing and web-based tools. Nucleic Acids Res.

[37] Clarke K, Gorley R (2015). PRIMER v7: user manual/tutorial.

[38] Clarke KR, Warwick RM (2001). Change in marine communities: an approach to statistical analysis and interpretation.

[39] Kruskal WH, Wallis WA (1952). Use of ranks in one-criterion variance analysis. J Am Stat Assoc.

[40] Anderson MJ (2001). A new method for non-parametric multivariate analysis of variance. Austral Ecol.

[41] Balk M, Keuskamp JA, Laanbroek HJ (2016). Potential for sulfate reduction in mangrove forest soils: comparison between two dominant species of the Americas. Front Microbiol.

[42] Schloss PD, Girard RA, Martin T (2016). Status of the archaeal and bacterial census: an update. MBio.

[43] Kim H, Ogram A, Bae HS (2017). Nitrification, Anammox and denitrification along a nutrient gradient in the Florida Everglades. Wetlands.

[44] Martens-Habbena W, Berube PM, Urakawa H (2009). Ammonia oxidation kinetics determine niche separation of nitrifying archaea and bacteria. Nature.

[45] French E, Bollmann A (2015). Freshwater ammonia-oxidizing archaea retain *amoA* mRNA and 16S rRNA during ammonia starvation. Life.

[46] Sorokin DY, Tourova TP, Kolganova TV (2002). *Thioalkalispira microaerophila* gen. Nov., sp. nov., a novel lithoautotrophic, sulfur-oxidizing bacterium from a soda lake. Int J Syst Evol Microbiol.

[47] Inagaki F, Takai K, Nealson KH, Horikoshi K (2004). *Sulfurovum lithotrophicum* gen. Nov., sp. nov., a novel sulfur-oxidizing chemolithoautotroph within the ε-Proteobacteria isolated from Okinawa Trough hydrothermal sediments. Int J Syst Evol Microbiol.

[48] Xiang X, Wang R, Wang H (2017). Distribution of Bathyarchaeota communities across different terrestrial settings and their potential ecological functions. Sci Rep.

[49] Stieglmeier M, Alves RJE, Schleper C (2014) The phylum thaumarchaeota. In: Rosenberg E, De Long E, Lory S, et al (eds) The prokaryotes. pp 347–362

[50] Xu Y, He Y, Tang X, Brookes PC, Xu J (2017). Reconstruction of microbial community structures as evidences for soil redox coupled reductive dechlorination of PCP in a mangrove soil. Sci Total Environ.

[51] Spang A, Saw JH, Jørgensen SL (2015). Complex archaea that bridge the gap between prokaryotes and eukaryotes. Nature.

[52] Inagaki F, Nunoura T, Nakagawa S (2006). Biogeographical distribution and diversity of microbes in methane hydrate-bearing deep marine sediments on the Pacific Ocean margin. Proc Natl Acad Sci USA.

[53] Andreote FD, Jiménez DJ, Chaves D (2012). The microbiome of Brazilian mangrove sediments as revealed by metagenomics. PLoS One.

[54] Oren A, Rosenberg E, De Long E, Lory S (2014). The family Xanthobacteraceae. The prokaryotes.

[55] Alongi DM (2005) Mangrove-microbe-soil relations. In: Kristensen E, Haese RR, Kostka JE (eds) Interactions between macro- and microorganisms in marine sediments. pp 85–103

[56] Reysenbach AL (2015) Thermoplasmatales ord. nov. In: Whitman WB (ed) Bergey’s Man. Syst. Archaea Bact. John Wiley & Sons, Inc. 10.1002/9781118960608.obm00055

[57] Otero XL, Lucheta AR, Ferreira TO (2014). Archaeal diversity and the extent of iron and manganese pyritization in sediments from a tropical mangrove creek (Cardoso Island, Brazil). Estuar Coast Shelf Sci.

[58] Rey JR, Carlson DB, Brockmeyer RE (2012). Coastal wetland management in Florida: environmental concerns and human health. Wetl Ecol Manag.

[59] Stringer CE, Rains MC, Kruse S, Whigham D (2010). Controls on water levels and salinity in a barrier island mangrove, Indian River Lagoon, Florida. Wetlands.

[60] Portnoy JW, Giblin AE (1997). Effects of historic tidal restrictions on salt marsh sediment chemistry. Biogeochemistry.

[61] Brockmeyer RE, Rey JR, Virnstein RW (1996). Rehabilitation of impounded estuarine wetlands by hydrologic reconnection to the Indian River Lagoon, Florida (USA). Wetl Ecol Manag.

