# Supplementary material for: Tide as Steering Factor in Structuring Archaeal and Bacterial Ammonia-Oxidizing Communities in Mangrove Forest Soils Dominated by Avicennia germinans and Rhizophora mangle
Source: Microb Ecol. 2017 Oct 23;75(4):997–1008. doi: 10.1007/s00248-017-1091-y (PMC5906487; doi:10.1007/s00248-017-1091-y)

Supplementary Material for:

Tide as steering factor in structuring archaeal and bacterial ammonia-oxidizing communities in mangrove forest soils dominated by *Avicennia germinans* and *Rhizophora mangle*

Magalí S. Marcos[1,2], Anthony Barboza[1,3], Rosalinde M. Keijzer[1], Hendrikus J. Laanbroek[1,4]*

[1] Department of Microbial Ecology, Netherlands Institute of Ecology (NIOO-KNAW), Wageningen, the Netherlands

[2] Laboratorio de Microbiología y Biotecnología, Instituto Patagónico para el Estudio de los Ecosistemas Continentales (IPEEC, CONICET), Puerto Madryn, Argentina

[3] Centro Interdisciplinar de Pesquisas em Biotecnologia – CIP-Biotec, Universidade Federal do Pampa, Campus São Gabriel, São Gabriel, Brazil

[4] Ecology and Biodiversity Group, Department of Biology, Utrecht University, Utrecht, the Netherlands

*Corresponding author: Hendrikus J. Laanbroek. Department of Microbial Ecology, Netherlands Institute of Ecology (NIOO-KNAW), P.O. Box 50, 6700 AB, Wageningen, the Netherlands. Phone: +31 (0)317 47 34 00. E-mail: r.laanbroek@nioo.knaw.nl

Table S1 Relative abundance (%) of bacterial genera in mangrove soils. Only genera above 1% are presented. Genera that mostly contributed to differences between mangrove species as determined by SIMPER analysis were highlighted in boldface

Phylum	Genus	Location and sample #																		
		PI-A			PI-R		SHI-A				SHI-R				NHI-A			NHI-R		
		2	3	4	3	4	1	2	3	4	1	2	3	4	1	3	4	1	2	4
Actinobacteria	Unclassified OM1 clade	3.3	2.6	2.0	1.1	1.0	1.2	1.8	1.5	2.2	4.2	2.5	3.8	2.7	1.8	3.1	3.3	2.4	1.4	1.5
	Mycobacterium	0.9	0.7	0.7	0.6	0.2	1.1	0.6	0.8	0.5	1.1	2.4	1.3	0.8	0.2	0.7	0.4	0.3	0.1	0.0
	Demequina	0.0	0.0	0.0	0.0	0.0	1.4	0.6	0.0	0.3	0.0	0.0	0.0	0.0	0.0	0.0	0.7	0.4	0.0	0.0
	Uncultured Elev-16S-1332	1.0	1.7	0.9	1.1	0.3	1.1	1.6	0.8	2.2	0.3	0.6	0.8	0.5	0.5	1.3	3.4	0.6	0.0	0.0
Bacteroidetes	*Gracilimonas*	0.0	0.0	0.0	0.0	0.0	0.0	0.0	0.0	0.0	0.0	0.0	0.0	0.0	0.0	1.6	0.0	0.0	0.0	0.0
	Uncultured Bacteroidetes VC2.1 Bac22	2.9	1.6	3.8	0.0	0.2	0.0	0.1	0.0	0.8	0.2	0.5	0.1	0.6	1.2	0.0	0.0	5.4	1.8	0.7
	Marinifilum	0.1	0.0	0.1	0.4	0.4	**0.0**	**0.0**	**0.0**	**0.2**	**0.3**	**0.3**	**8.8**	**2.7**	0.1	0.0	0.0	0.5	0.2	0.6
	Uncultured Marinilabiaceae	0.5	0.2	0.3	1.8	1.4	0.0	0.0	0.0	0.1	0.4	0.1	0.0	0.1	0.9	0.5	0.0	0.5	0.0	0.5
	Lutibacter	0.0	0.0	0.0	0.0	0.0	0.0	0.0	1.3	2.2	0.1	0.2	0.3	0.0	0.1	0.0	0.0	2.0	0.1	0.0
	Tenacibaculum	0.0	0.0	0.0	0.0	0.0	**16.9**	**0.0**	**0.0**	**0.0**	**0.0**	**0.0**	**0.0**	**0.0**	0.0	0.1	0.0	0.0	0.0	0.0
Chloroflexi	**Uncultured Anaerolineaceae**	**1.3**	**2.9**	**3.0**	**4.6**	**10.7**	1.0	1.2	1.0	1.5	2.6	0.4	1.3	5.5	5.3	2.8	15.6	2.2	0.9	0.5
	Uncultured Ardenticatenia	0.1	0.1	0.0	0.2	0.3	0.0	0.1	0.0	0.0	0.0	0.0	0.0	0.0	0.3	0.1	4.0	0.2	0.1	0.2
Firmicutes	*Aeribacillus*	0.0	0.0	0.0	0.0	0.0	0.0	0.0	0.0	0.0	0.0	0.1	0.0	0.0	0.0	0.9	0.0	0.0	0.1	3.3
	Halobacillus	0.1	0.0	0.0	0.0	0.0	0.0	0.1	0.0	0.1	0.1	0.1	0.1	0.1	0.3	0.3	1.1	0.4	0.1	0.5
	Sedimentibacter	0.0	0.0	0.0	0.0	0.0	0.0	0.2	0.0	0.1	0.0	0.0	1.2	0.0	0.0	0.0	0.0	0.0	0.0	0.0
	Fusibacter	0.0	2.1	0.4	0.0	0.2	0.1	2.2	0.2	3.2	0.6	0.5	0.2	0.4	8.7	7.1	0.0	0.9	0.2	2.1
	Uncultured Gracilibacteraceae	0.0	0.0	0.0	0.0	0.0	0.0	0.0	0.0	0.0	0.1	0.2	2.0	0.7	0.0	0.0	0.0	0.0	0.0	0.0
Gemmatimonadetes	Uncultured BD2-11 terrestrial group	1.3	3.2	1.5	0.9	1.1	0.9	1.7	1.1	0.6	0.8	0.5	0.9	0.9	1.1	1.1	1.7	1.0	0.6	1.2
	Uncultured PAUC43f marine benthic group	0.2	0.5	0.2	0.0	0.0	0.5	1.5	0.2	0.2	0.5	0.3	0.3	0.3	0.2	0.2	0.3	0.2	0.1	0.1
Nitrospirae	Uncultured Nitrospiraceae	0.0	0.1	0.1	1.7	1.8	0.0	0.1	0.0	0.0	0.3	0.1	0.3	0.3	0.3	0.1	0.2	0.1	0.0	0.3
Parcubacteria	Uncultured Parcubacteria	0.0	0.0	0.0	0.0	0.1	0.0	0.0	0.0	0.0	0.0	0.0	0.0	0.0	2.3	0.0	0.0	0.0	0.0	0.0
Planctomycetes	Pir4 lineage	0.8	0.3	0.9	0.7	0.4	1.1	1.4	1.9	2.0	0.9	0.5	0.6	1.0	1.6	1.5	1.0	1.9	0.0	0.2

	Planctomyces	0.3	0.2	0.3	0.3	0.3	0.7	0.9	0.8	1.0	0.5	0.5	0.3	0.6	1.1	0.7	0.6	1.2	0.1	0.8
	Uncultured Planctomycetaceae	0.3	0.1	0.5	0.5	0.3	0.7	1.4	0.7	0.8	0.5	0.1	0.2	0.6	0.9	0.9	0.6	0.9	0.0	0.4
Proteobacteria	*Filomicrobium*	0.2	0.2	0.2	0.1	0.1	0.4	0.2	0.4	0.3	1.5	1.1	1.0	1.0	0.2	0.4	0.4	1.2	0.5	0.8
Proteobacteria	Uncultured JG34-KF-361	1.3	1.3	0.9	0.7	0.6	1.0	1.2	0.8	1.1	0.1	0.0	0.4	0.2	0.5	0.2	0.4	0.4	0.2	0.1
	Hoeflea	0.1	0.0	0.1	0.1	0.1	0.3	0.3	0.3	0.2	0.5	0.5	0.3	0.3	0.7	1.5	0.3	0.4	0.8	0.5
	Unclassified Phyllobacteriaceae	0.3	0.2	0.2	0.2	0.3	1.8	0.6	0.6	0.5	0.5	0.3	0.3	0.3	0.2	0.3	0.3	0.8	0.9	0.3
	Rhizobium	0.1	0.0	0.0	0.0	0.0	3.0	0.2	0.0	0.1	0.0	0.0	0.0	0.0	0.0	0.1	0.0	0.1	0.0	0.0
	Bauldia	0.4	0.4	0.3	0.5	0.5	0.8	1.1	0.5	0.6	0.1	0.1	0.1	0.1	0.1	0.3	0.2	0.3	0.3	0.2
	Unclassified Rhizobiales Incertae Sedis	0.6	1.1	0.5	0.5	0.6	0.7	1.6	0.3	0.5	0.1	0.0	0.2	0.2	0.2	0.1	0.3	0.1	0.0	0.1
	Parvibaculum	0.0	0.0	0.0	0.0	0.0	2.8	0.0	0.0	0.1	0.0	0.1	0.0	0.0	0.0	0.0	0.4	0.1	0.1	0.0
	Uncultured Rhodobiaceae	0.5	0.4	0.2	0.1	0.1	0.7	1.0	0.9	1.0	0.3	0.1	0.6	0.6	0.2	0.3	0.3	0.8	0.5	0.3
	Unclassified Rhodobiaceae	1.1	0.9	0.7	0.9	0.6	2.0	2.1	2.1	2.0	2.4	1.7	1.5	1.8	0.8	0.9	1.8	2.5	0.8	1.5
	Pseudolabrys	0.9	0.8	0.6	0.5	0.7	**3.0**	**5.1**	**2.5**	**3.0**	**0.3**	**0.6**	**0.7**	**0.6**	0.3	0.5	0.6	1.4	0.6	0.3
	Variibacter	0.2	0.4	0.3	0.8	1.2	1.7	0.9	0.8	0.7	0.3	0.3	0.4	0.1	0.0	0.0	0.1	0.7	0.1	0.1
	Unclassified Rhodobacteraceae	0.3	0.2	0.3	0.3	0.4	0.2	0.6	0.3	0.3	1.0	0.5	0.6	0.7	0.2	0.5	2.7	0.6	0.4	0.7
	Uncultured MSB-1E8	0.1	0.4	0.1	0.2	0.1	0.0	0.1	0.1	0.2	0.5	0.1	0.2	0.4	0.7	1.5	1.0	0.6	0.1	0.4
	Thalassospira	0.0	0.1	0.1	0.0	0.0	0.0	0.0	0.0	0.0	0.0	0.0	0.0	0.0	0.0	0.7	1.6	0.6	0.0	0.0
	Uncultured Rhodospirillaceae	1.6	2.4	1.6	2.1	2.3	1.5	2.6	2.0	2.2	1.9	1.3	1.7	1.6	1.9	2.1	1.2	2.6	1.2	2.5
	Uncultured Nitrosomonadaceae	0.0	0.0	0.0	1.6	2.5	0.1	0.2	0.0	0.1	0.1	0.0	0.1	0.1	0.0	0.0	0.0	0.1	0.0	0.2
	Candidatus Accumulibacter	0.0	0.0	0.0	0.0	0.0	0.0	0.0	0.0	0.8	0.0	0.0	2.9	0.0	0.0	0.0	0.0	0.0	0.0	0.0
	Uncultured Bacteriovoracaceae	0.2	0.6	0.3	1.0	1.2	0.1	0.1	0.0	0.1	0.2	0.2	0.7	0.3	0.2	0.0	0.3	0.2	0.0	0.0
	Desulfobacterium	0.0	0.5	1.4	0.8	0.1	0.0	0.0	0.0	0.1	0.4	0.1	1.1	0.7	1.0	0.1	0.0	2.0	0.9	0.6
	Desulfotignum	0.0	0.0	0.0	0.1	0.1	0.0	0.0	0.0	0.0	0.2	0.2	0.0	0.3	1.4	0.0	0.0	0.2	0.0	0.0
	SEEP-SRB1	0.4	1.1	0.6	0.3	0.6	0.7	1.0	0.9	0.6	1.3	0.5	0.7	0.8	0.3	0.5	0.5	0.7	0.5	0.7
	Sva0081 sediment group	0.7	1.8	0.8	0.7	0.7	0.5	1.0	0.8	0.4	1.5	0.9	0.8	0.9	1.6	1.5	0.9	1.1	0.5	1.3
	Desulfobulbus	0.0	0.0	0.1	0.6	1.4	0.1	0.0	0.1	0.1	1.1	0.3	0.4	0.5	0.1	1.5	0.1	0.8	0.2	0.6
	H16	0.1	0.1	0.1	1.4	1.4	0.3	0.5	0.3	0.7	0.2	0.0	0.2	0.2	0.1	0.1	0.0	0.3	0.1	0.3
	Desulfuromusa	0.2	0.1	0.2	0.4	0.2	0.4	0.0	0.0	1.4	0.1	0.1	0.6	0.3	0.1	0.1	0.0	0.0	0.0	0.0
	Uncultured Sandaracinaceae	0.3	0.5	0.5	0.6	0.5	0.5	1.0	0.7	0.5	1.3	0.9	0.8	0.7	1.1	1.0	0.6	0.9	0.5	1.2
	Uncultured NB1-j	1.0	2.5	1.4	1.5	1.6	0.9	2.0	1.6	1.3	1.5	0.7	1.6	1.0	0.8	1.8	1.4	1.3	0.4	2.1
	Uncultured Syntrophobacteraceae	0.5	1.1	1.2	0.7	1.1	0.4	1.4	0.8	0.8	1.9	0.6	1.3	1.4	0.4	0.8	0.6	0.6	0.2	0.7

	Arcobacter	0.4	1.2	0.1	0.2	0.1	**0.2**	**0.7**	**29.3**	**1.1**	**0.0**	**0.1**	**0.1**	**0.3**	0.0	0.0	0.0	1.0	0.0	0.4
	Sulfurimonas	2.9	1.3	1.7	3.3	0.1	**0.0**	**0.0**	**0.0**	**4.8**	**0.3**	**0.5**	**1.2**	**0.9**	3.1	2.0	0.0	0.4	0.6	1.5
	Sulfurovum	**43.8**	**20.1**	**38.2**	**1.6**	**0.1**	**0.0**	**0.0**	**0.0**	**16.1**	**0.1**	**0.1**	**0.0**	**0.1**	9.7	0.2	0.0	0.2	43.8	8.1
	Marinobacter	0.0	0.0	0.3	0.0	0.0	0.0	0.1	0.1	0.1	0.2	0.0	0.0	0.0	0.2	3.7	2.5	0.1	0.2	0.6
Proteobacteria	*Idiomarina*	0.0	0.0	0.0	0.0	0.0	0.0	0.0	0.0	0.0	0.0	0.0	0.0	0.0	0.0	0.1	1.6	0.0	0.0	0.0
	Uncultured BD7-8 marine group	0.7	0.3	1.4	0.1	0.1	0.1	0.3	0.4	0.3	0.6	1.0	0.3	0.5	1.0	0.6	0.1	1.3	0.3	0.1
	Thioalkalispira	**0.3**	**0.1**	**1.5**	**6.4**	**4.1**	**0.1**	**0.1**	**0.1**	**0.3**	**1.4**	**3.1**	**10.4**	**4.2**	2.5	0.2	0.2	3.0	0.3	6.4
	Thiogranum	0.1	0.0	0.1	0.1	0.1	0.3	0.2	0.1	0.2	0.7	0.5	0.3	0.4	0.1	1.0	0.1	0.5	1.2	1.2
	Sedimenticola	0.0	0.0	0.0	0.0	0.0	**0.0**	**0.0**	**0.0**	**0.0**	**2.5**	**33.4**	**1.2**	**12.6**	0.0	0.0	0.0	0.7	0.1	0.0
	Uncultured Gammaproteobacteria Incertae Sedis	0.2	0.2	0.3	0.3	0.4	0.5	0.6	0.3	0.3	1.0	0.9	0.5	0.5	0.3	0.9	0.2	0.8	0.6	1.3
	Halomonas	0.0	0.4	0.0	0.0	0.0	0.9	0.0	0.0	0.0	0.0	0.0	0.0	0.0	0.1	0.4	2.8	0.1	0.4	1.7
	Amphritea	0.4	1.2	0.3	0.7	0.3	0.0	0.0	0.0	0.0	0.0	0.1	0.0	0.1	0.0	0.1	0.0	1.1	1.4	1.5
	Uncultured Piscirickettsiaceae	0.0	0.0	0.0	0.0	0.1	0.0	0.0	0.0	0.0	0.0	0.0	0.0	0.0	0.0	0.0	0.0	0.0	1.1	0.0
	Uncultured Thiotrichaceae	0.1	0.2	0.0	0.6	0.2	3.6	0.1	0.0	0.0	0.8	0.3	0.4	0.5	0.1	0.9	0.0	0.2	0.5	1.2
	Vibrio	0.0	0.2	0.1	1.0	0.0	0.0	0.1	5.2	0.1	0.1	0.0	0.2	0.1	0.1	0.0	0.0	0.1	0.2	0.4
	Uncultured JTB255 marine benthic group	0.5	0.7	0.6	0.7	0.2	0.1	0.2	0.1	0.1	1.2	1.3	0.7	0.6	0.8	1.9	0.6	0.5	0.3	1.1
	Unclassified JTB255 marine benthic group	1.4	2.6	1.7	0.9	1.3	1.3	2.9	1.6	2.1	4.1	2.3	2.4	1.9	0.4	1.4	0.2	1.7	1.1	2.2
	Unclassified Xanthomonadales	0.7	0.8	0.8	1.4	1.6	0.7	1.5	1.4	1.1	2.1	1.3	1.1	0.8	0.3	0.7	0.3	0.7	0.1	0.1
	Luteibacter	0.0	0.0	0.0	0.0	0.0	1.9	0.0	0.0	0.0	0.0	0.0	0.0	0.0	0.0	0.0	4.2	0.0	0.0	0.0
	Unclassified Xanthomonadaceae	0.0	0.0	0.0	0.0	0.0	4.7	0.4	0.0	0.0	0.0	0.0	0.0	0.0	0.0	0.0	0.0	0.0	0.0	0.0
	Uncultured Gammaproteobacteria	0.1	0.1	0.1	3.2	1.4	0.5	0.5	0.3	0.7	1.7	0.7	1.1	1.0	0.2	1.1	0.5	1.2	1.1	2.2
Spirochaetae	*Spirochaeta 2*	0.6	1.2	0.9	1.0	1.9	0.4	0.5	0.4	0.4	2.5	1.0	1.1	2.5	0.6	0.2	0.8	0.8	0.2	0.9

Table S2 Relative abundance (%) of archaeal genera in mangrove soils. Only genera above 1% are presented. Genera that mostly contributed to differences between mangrove species as determined by SIMPER analysis were highlighted in boldface

		Location and sample #																		
Phylum	Genus	PI-A			PI-R		SHI-A				SHI-R				NHI-A			NHI-R		
		2	3	4	3	4	1	2	3	4	1	2	3	4	1	3	4	1	2	4
Aenigmarchaeota	*Candidatus Aenigmarchaeum*	0.4	0.0	0.0	0.0	0.0	3.9	2.8	3.6	0.9	0.0	0.0	0.0	1.8	1.4	0.0	0.0	0.6	0.0	0.0
AK8	Uncultured AK8	0.4	0.0	0.0	0.0	0.1	0.0	0.0	0.0	0.0	0.0	0.0	1.0	0.9	0.0	0.0	0.0	0.0	0.0	0.0
Altiarchaeales	Uncultured Altiarchaeales	0.0	0.0	0.0	1.5	1.1	0.0	0.0	0.0	0.0	0.0	0.0	0.0	0.0	0.0	0.0	0.0	0.0	0.0	0.0
Bathyarchaeota	**Uncultured Bathyarchaeota**	**77.3**	**53.1**	**57.5**	**33.7**	**32.2**	**68.8**	**80.2**	**44.8**	**45.0**	**19.5**	**30.0**	**50.0**	**33.3**	42.9	20.3	40.9	21.3	26.7	20.0
Euryarchaeota	*Haladaptatus*	0.0	0.0	0.0	0.0	0.0	0.0	0.0	0.0	0.0	0.0	0.0	0.0	0.5	2.9	1.6	8.8	0.9	0.0	0.0
	Halogeometricum	0.4	0.0	0.0	0.0	0.0	6.5	0.5	0.0	1.3	0.0	0.0	0.0	0.0	0.0	0.0	5.6	0.0	0.0	0.0
	Halohasta	0.0	0.0	0.0	0.0	0.0	0.0	0.0	0.0	0.0	0.0	0.0	0.0	0.0	0.0	0.0	0.0	0.0	6.7	0.0
	Halolamina	0.0	0.0	0.0	0.0	0.0	1.3	0.0	0.0	0.0	0.0	0.0	0.0	0.0	0.0	0.0	3.2	0.0	0.0	0.0
	Halomarina	0.0	0.0	0.0	0.0	0.0	0.0	0.5	0.0	0.0	0.0	0.0	0.0	0.0	1.4	0.0	5.1	0.3	0.0	0.0
	Halorussus	0.0	0.0	0.0	0.0	0.0	0.0	0.0	0.0	0.0	0.0	0.0	0.0	0.0	0.0	0.0	3.2	0.3	0.0	0.0
	Natronomonas	0.0	0.0	0.0	0.0	0.0	0.0	0.0	0.0	1.3	0.0	0.0	0.3	0.0	0.0	0.0	0.3	0.0	0.0	0.0
	Uncultured Halobacteriaceae	0.4	0.0	0.0	0.0	0.0	0.0	0.0	0.0	0.4	0.0	5.0	0.3	0.0	0.0	0.0	2.3	0.0	0.0	3.3
	Unclassified Halobacteriaceae	1.2	1.0	0.0	0.0	0.0	0.0	0.5	0.6	0.9	2.4	0.0	0.0	0.9	0.0	1.6	5.6	0.0	0.0	0.0
	Methanobacterium	0.0	0.0	0.0	1.0	1.4	0.0	0.0	0.0	0.0	0.0	0.0	0.0	0.0	0.0	0.0	0.0	0.0	0.0	0.0
	Uncultured Methanomicrobiaceae	0.0	0.0	0.0	2.9	0.8	0.0	0.0	0.0	0.0	0.0	0.0	0.0	0.0	0.0	0.0	0.0	0.0	0.0	0.0
	Methanolinea	0.0	0.0	0.0	1.5	1.5	0.0	0.0	0.0	0.0	0.0	0.0	0.0	0.0	0.0	0.0	0.0	0.0	0.0	0.0
	Methanosaeta	0.0	0.0	0.0	2.9	2.5	0.0	0.0	0.0	0.0	0.0	0.0	0.0	0.0	0.0	0.0	0.0	0.0	0.0	0.0
	Methanococcoides	0.0	0.0	0.0	0.0	0.0	**0.0**	**0.0**	**0.0**	**0.0**	**0.0**	**0.0**	**22.6**	**0.9**	1.4	0.0	0.0	0.0	0.0	0.0
	Methanolobus	0.0	1.0	0.0	0.5	0.1	0.0	0.0	0.0	0.0	0.0	0.0	0.0	0.0	0.0	0.0	0.1	0.0	6.7	10.0
	Methanosarcina	0.0	0.0	0.0	0.0	0.0	0.0	0.0	0.0	0.0	0.0	0.0	3.8	0.0	0.0	0.0	0.0	0.0	0.0	0.0
	Uncultured 20c-4	0.0	0.0	0.0	0.0	0.0	0.0	0.0	0.0	0.0	0.0	0.0	0.3	1.4	0.0	0.0	0.1	0.0	0.0	0.0
	**Uncult. Marine Benthic Group D - DHVEG-1**	0.0	7.3	13.1	0.0	0.3	**0.0**	**0.0**	**0.6**	**0.0**	**29.3**	**10.0**	**3.8**	**21.9**	4.3	0.0	3.1	0.0	0.0	0.0

	**Unclas. Marine Benthic Group D - DHVEG-1**	0.4	1.0	5.6	1.0	0.0	**0.0**	**0.0**	**0.6**	**0.0**	**7.3**	**10.0**	**1.7**	**7.8**	1.4	0.0	1.8	0.0	0.0	0.0
Lokiarchaeota	**Uncultured Lokiarchaeota**	**7.4**	**14.6**	**11.3**	**0.0**	**1.7**	6.5	3.3	1.8	3.1	2.4	0.0	2.8	9.1	4.3	0.0	13.0	1.8	10.0	3.3
MHVG[1]	Uncultured MHVG	0.0	0.0	0.6	0.0	0.0	0.0	0.0	0.0	0.0	0.0	0.0	1.7	0.0	0.0	0.0	0.0	0.0	0.0	0.0
MEG[2]	Uncultured MEG	0.0	0.0	0.0	1.0	0.1	0.0	0.0	1.2	0.0	0.0	0.0	0.3	0.9	0.0	0.0	0.0	0.0	0.0	0.0
Thaumarchaeota	**Uncultured Marine Group I**	**0.4**	**0.0**	**0.0**	**34.6**	**31.3**	**0.0**	**0.0**	**1.2**	**0.0**	**4.9**	**0.0**	**0.7**	**0.5**	5.7	10.9	0.0	8.4	0.0	10.0
	**Unclassified Marine Group I**	**0.0**	**0.0**	**0.0**	**3.4**	**10.3**	0.0	0.5	0.6	0.9	0.0	0.0	0.0	0.0	0.0	0.0	0.0	0.0	0.0	0.0
	Uncultured Soil Crenarchaeotic Group (SCG)	0.0	0.0	0.0	0.0	0.2	0.0	0.0	0.6	0.4	0.0	0.0	0.7	0.0	0.0	1.6	0.1	3.9	3.3	0.0
Uncultured	Uncultured Archaea	0.8	3.1	2.5	2.4	1.8	0.0	1.4	0.6	1.3	12.2	10.0	0.7	1.8	0.0	1.6	0.6	0.3	3.3	0.0
Woesearchaeota	Uncultured Woesearchaeota	0.8	4.2	1.9	0.5	2.1	0.0	0.0	1.2	15.3	0.0	0.0	2.1	4.1	8.6	0.0	0.3	0.0	0.0	0.0

[1]MHVG, Marine Hydrothermal Vent Group

[2]MEG, Miscellaneous Euryarchaeotic Group

Table S3 Physico-chemical properties of soil samples. Presented are the values of combined soil samples per sampling location

Soil properties	Sample						Reference
	PI-A	PI-R	SHI-A	SHI-R	NHI-A	NHI-R	
Particle size (DV50)[1]	84	87	92	116	157	292	[1]
TOC (%)	26	22	26	13	3.5	6.1	[1]
pH	7.7	7.4	5.7	7.4	7.5	6.7	[1]
Salinity (g L^{-1})	69	39	41	37	95	57	[1]
Nitrate (g kg^{-1})	nd[2]	nd[2]	nd[2]	nd[2]	nd[2]	nd[2]	[1]
Nitrite (g kg^{-1})	nd[2]	nd[2]	nd[2]	nd[2]	nd[2]	nd[2]	[1]
Sulfate (g kg^{-1})	15.3	10.7	9.6	2.7	3.4	2.2	This study
Sulfur (g kg^{-1})	431	12	406	233	2.9	1.7	This study
Aluminum [Al] (mg kg^{-1})	9,480	40,000	25,500	19,700	9,330	8,380	This study
Arsenic [As] (mg kg^{-1})	< 5	< 5	< 5	< 8.9	< 5	< 5	This study
Cadmium [Cd] (mg kg^{-1})	0.2	< 0.1	< 0.1	< 0.2	< 0.1	< 0.1	This study
Calcium [Ca] (mg kg^{-1})	17,400	12,100	7,780	5,910	17,700	2,330	This study
Chromium [Cr] (mg kg^{-1})	29	110	42	30	8.7	7.7	This study

Cobalt [Co] (mg kg^{-1})	1.6	6.3	4.1	3	0.99	1.3	This study
Phosphorus [P] (mg kg^{-1})	1,090	1,140	827	556	756	168	This study
Iron [Fe] (mg kg^{-1})	3,540	12,200	8,190	8,290	2,630	3,880	This study
Potassium [K] (mg kg^{-1})	5,600	5,800	6,500	6,600	5,000	4,000	This study
Copper [Cu] (mg kg^{-1})	3.9	8.9	24	11	1.8	2.8	This study
Mercury [Hg] (mg kg^{-1})	< 0.1	< 0.1	< 0.1	< 0.1	< 0.1	< 0.11	This study
Lead [Pb] (mg kg^{-1})	1.9	11	12	11	14	3.5	This study
Magnesium [Mg] (mg kg^{-1})	15,000	14,000	11,000	6,400	2,700	2,200	This study
Manganese [Mn] (mg kg^{-1})	16	64	38	37	34	40	This study
Sodium [Na] (mg kg^{-1})	74,000	61,000	52,000	27,000	16,000	12,000	This study
Nickel [Ni] (mg kg^{-1})	14	28	16	8	3.6	3.2	This study
Zinc [Zn] (mg kg^{-1})	39	53	47	33	8.8	12	This study

[1]As volume based mean diameter

[2]Not detectable (< 0.5 mg per kg dry soil)

Reference

1. Balk M, Laverman AM, Keuskamp JA, Laanbroek HJ (2015) Nitrate ammonification in mangrove soils: A hidden source of nitrite? Front Microbiol. doi: 10.3389/fmicb.2015.00166

Fig. S1 Effect of mangrove tree species (*Avicennia germinans* vs. *Rhizophora mangle*) on the community composition of AOA and AOB, based on PCR-DGGE of *amoA* genes

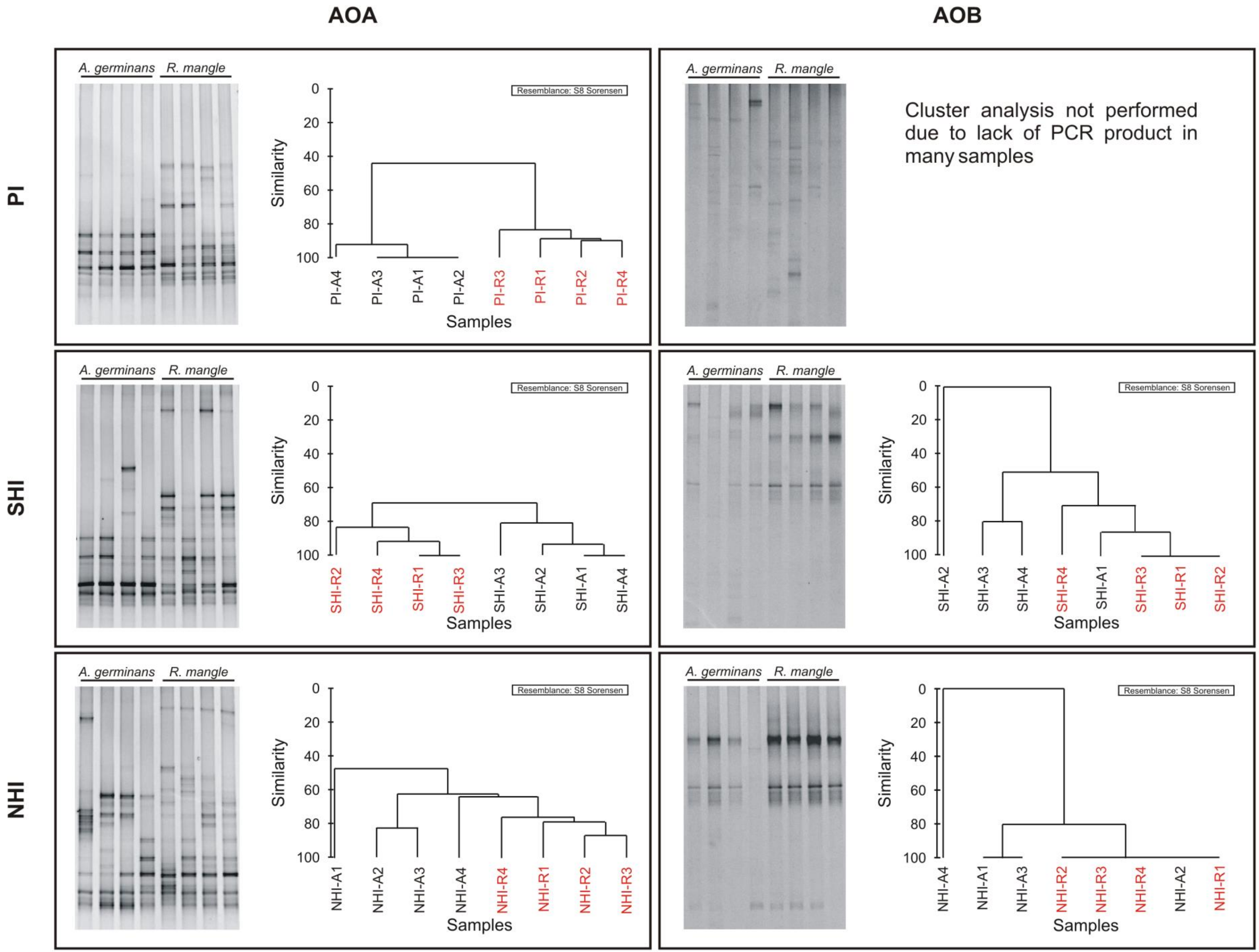

Fig. S2 Cluster analysis of soil samples covered by *Avicennia germinans* and *Rhizophora mangle* based on the relative abundance of OTUs classified as Nitrosomonadaceae

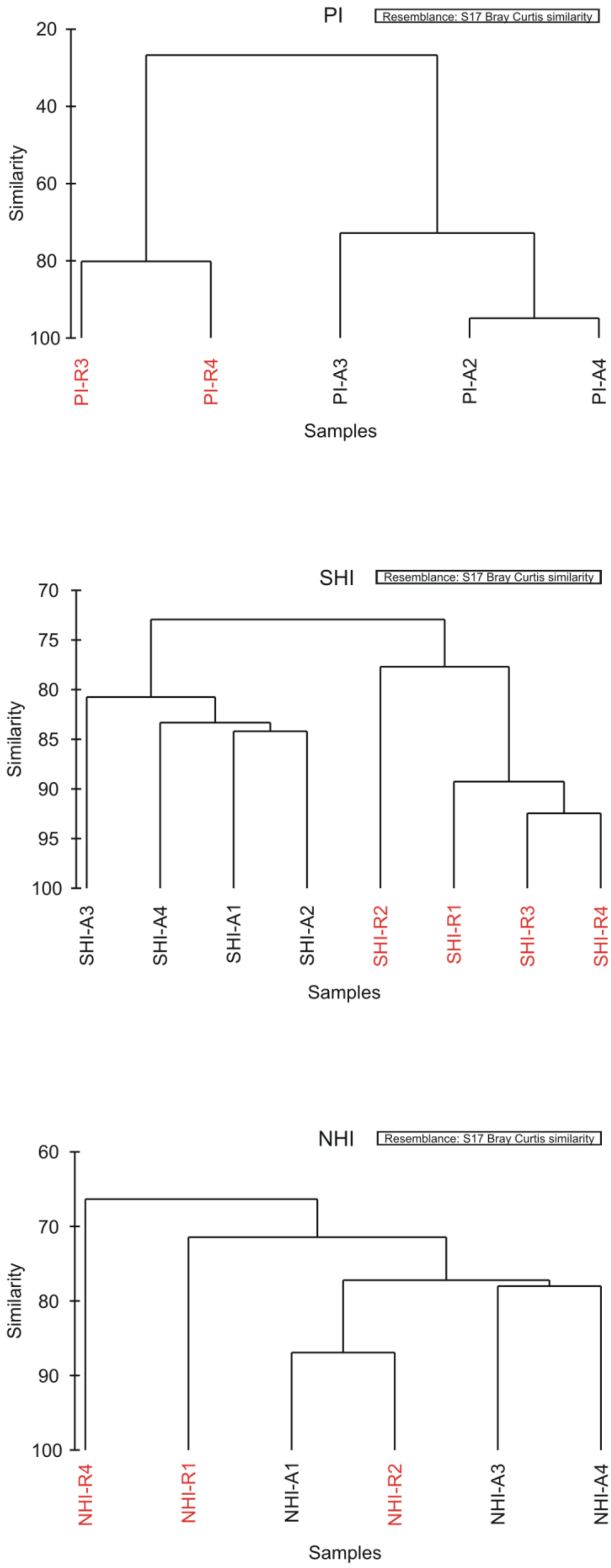

Fig. S3 Principal component analysis of the physico-chemical properties of mangrove soil samples. Vectors in blue represent physico-chemical properties of the soils

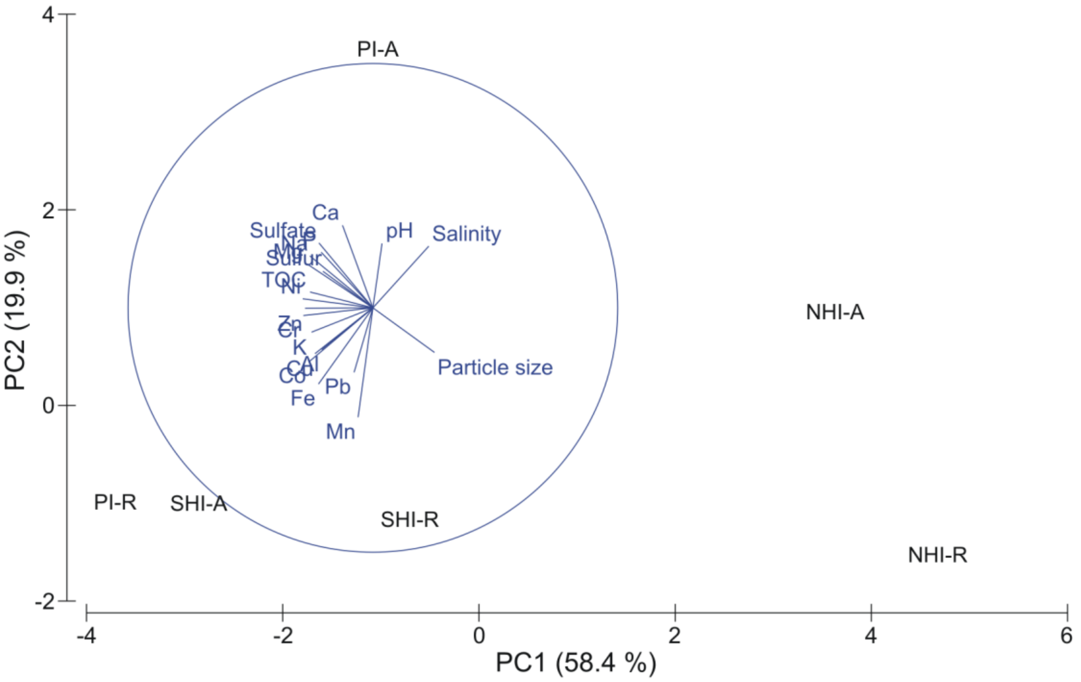

Supplement: Supplementary file 1 — (PDF 911 kb) [file 248_2017_1091_MOESM1_ESM.pdf]
